# Concept Drift Mitigation in Low-Cost Air Quality Monitoring Networks

**DOI:** 10.3390/s24092786

**Published:** 2024-04-27

**Authors:** Gerardo D’Elia, Matteo Ferro, Paolo Sommella, Sergio Ferlito, Saverio De Vito, Girolamo Di Francia

**Affiliations:** 1 TERIN-SSI-EDS Laboratory, ENEA CR-Portici, P. le E. Fermi 1, 80055 Portici, Italy; sergio.ferlito@enea.it (S.F.); saverio.devito@enea.it (S.D.V.); girolamo.difrancia@enea.it (G.D.F.); 2 Department of Industrial Engineering (DIIn), University of Salerno, Via Giovanni Paolo II, 132, 84084 Fisciano, Italy; psommella@unisa.it; 3 Hippocratica Imaging S.r.l., Via Giulio Pastore, 32, 84131 Salerno, Italy; mferro@hippocratica-imaging.it

**Keywords:** air quality network, concept drift, general calibration, global calibration, importance weighting, relative expanded uncertainty, calibration model update

## Abstract

Future air quality monitoring networks will integrate fleets of low-cost gas and particulate matter sensors that are calibrated using machine learning techniques. Unfortunately, it is well known that concept drift is one of the primary causes of data quality loss in machine learning application operational scenarios. The present study focuses on addressing the calibration model update of low-cost NO_2_ sensors once they are triggered by a concept drift detector. It also defines which data are the most appropriate to use in the model updating process to gain compliance with the relative expanded uncertainty (REU) limits established by the European Directive. As the examined methodologies, the general/global and the importance weighting calibration models were applied for concept drift effects mitigation. Overall, for all the devices under test, the experimental results show the inadequacy of both models when performed independently. On the other hand, the results from the application of both models through a stacking ensemble strategy were able to extend the temporal validity of the used calibration model by three weeks at least for all the sensor devices under test. Thus, the usefulness of the whole information content gathered throughout the original co-location process was maximized.

## 1. Introduction

Scientific and technical communities have long recognized the potential of exploiting low-cost sensors in modern air quality monitoring networks [1]. The main derived advantage is the possibility of deploying a significant number of sensing nodes at reasonable costs while densifying the sparse regulatory monitoring network. This in turn allows for long-sought high-density assessments of air pollution phenomena known to exhibit inherent high spatio-temporal variance [2]. The final outcomes include enhanced awareness, focused, efficient, and shared remediation policies, improving urban planning and reducing environmental and health inequalities. However, the large-scale application of such a technology is still hindered by inherent data quality issues. Correcting sensors’ sensitivity to non-target gases and environmental variables is mandatory to achieve the needed accuracy [3]. Moreover, the deterioration in the quality of the measurements occurring during long-term operational deployment decreases the data quality perception for these devices. Often, before any real-life deployment, a costly co-location time at certified reference stations is needed to obtain sufficient data to derive sensor calibration functions. The calibration obtained in the controlled environment failed to provide the requested robustness. Unfortunately, sensor fabrication variance forces this process to be repeated for each and any sensor by determining overall high costs and logistic bottlenecks. Moreover, every ab initio-derived calibration function will fail in the long term due to changes in sensor characteristics like poisoning or ageing [4].

Machine learning (ML) algorithms are recognized as a very convenient methodology to derive calibration functions [5]. Unfortunately, due to the operational scenario in which the sensor is forced to operate, the well-known independent and identically distributed samples hypothesis (*i.i.d. assumption*) is certainly violated. Indeed, the calibrated devices will have to operate in different conditions from those learned during the short-term co-location phase. Factors like seasonal environmental variations, anthropogenic forces, micrometeorology-related fluctuations in pollutant concentration levels, sensor aging, and so on will determine significant changes in the ongoing operative conditions [6]. As such, the data distribution model is a paramount issue [7]. The change in the type (and its location and spread parameters) of distribution will negatively impact the accuracy of the model output [8]. When this condition occurs, it defines the presence of a *concept drift* [9,10]. Concept drift is in fact a condition in which the statistical distribution of the target and input variables of a machine learning model has changed or continuously changed over time. The analysis of concept drift occurrence has given rise to the need for machine learning algorithms that are capable to learn in so-called “non-stationary environments” [6].

How to efficiently update the calibration model, after a concept drift condition has been recognized, is still an open question. Remote calibration and hierarchical network management are two of the most often proposed approaches.

With the term *Remote Calibration*, researchers indicate continuous or repeated recalibration schemes relying on a reference data stream from remote stations exploiting particular conditions and hypotheses. After a seminal paper by Tsujita et al. in 2005, different teams involved in air quality monitoring using low-cost sensor technologies have recently suggested that data coming from remote regulatory grade stations should be exploited, in low-spatial-variance conditions, to correct the effects of a drifting calibration in low-cost air quality networks [11]. It is actually reasonable to suppose a uniform spatial distribution of the pollutant concentration in a restricted geographical area at certain times when local emissions are negligible [12,13]. Based on this hypothesis and, therefore, thanks to data from a nearby regulatory station, in the case of uniformly low pollutant concentrations (e.g., during early morning hours), it could be possible to correct the baseline response of targeted gas sensors [14]. However, the generalization of such encouraging method findings has to be carefully evaluated since the spatial and temporal variance of the recorded phenomena may be significantly different depending on the geographic context negatively affecting the performance of remote calibration approaches [4].

Hierarchical networks, including “golden” reference stations (proxies), on top have also been proposed as a solution to the problem of the continuous calibration of low-cost air quality monitoring systems (LCAQMSs). Usually, regulatory instrumentations are on top in hierarchy, while some well-calibrated low-cost nodes are also entered into intermediate positions. These latter nodes which are limited in number can be regularly recalibrated, offering a quasi-gold reference data stream which can be used to continuously recalibrate other low-cost nodes. This can be achieved, for example, by regularly collocating these nodes with the latter by relocating the proxy nodes for short calibration-oriented periods. Alternatively, by carefully designing node deployment, proxy measurements can be used to remotely adjust the calibration of the low-cost nodes. One notable example is the Breathe London project, in which quasi-golden node data streams are exploited remotely [15]. Another way to practically implement this is by matching the mean and standard deviations between the measures of the proxy itself and low-cost sensor data over a fixed time window [16]. Based on this methodology, the Aeroqual Inc. company (Auckland, New Zealand) has developed a virtual calibration service for its own products (www.aeroqual.com (accessed on 23 April 2024)).

At present, continuous calibration strategies exploiting remote data are feasible and cost-effective procedures for continuous recalibration, keeping the required accuracy level for long-term deployments. Hourly averaged pollution data are, in fact, frequently published by regulatory institutions for the convenience of citizens and other users. This means that such data are reachable for everyone through API REST services. One possible implementation to be considered is MOMA (the acronym for MOment MAtching), which is the commercial name of the virtual calibration service distributed by the Aeroqual Inc. company. This service updates the calibration at fixed time intervals at an early development stage [17]. To apply a similar workflow, we took such a service into consideration and explored the possibility of triggering calibration by updating using a concept drift detector. This methodology will allow for the performance of recalibration only when specifically needed, saving precious computational resources and avoiding unwanted overfitting errors.

In order to select the suitable strategies to mitigate the effects of the concept drift, the following three main steps have been carried out: (i) understanding which data should be used to update the model; (ii) evaluation and validation of the general calibration model and importance weighting calibration model in the presence of a concept drift; (iii) attempting to improve the quality of pollution estimations data with the stacking ensemble technique in air quality network scenarios.

## 2. Materials and Methods

### 2.1. Experimental Campaign

The LCAQMS employed is based on an electrochemical sensors array using Alphasense (Braintree, UK) A4 class sensor units, respectively, targeted to carbon monoxide (CO-A4), nitrogen dioxide (NO2-A43F), and ozone (O3-A431). Relative humidity (RH) and temperature (T) sensors complete the sensing array. The electrochemical gas sensors array is mounted on an analog front-end provided by Alphasense that outputs the signals related to the concentration of monitored air gases. The working electrode (WE) and the auxiliary electrode (AE) signals of each type of sensor are acquired and converted by an ST Microelectronics (Geneva, Switzerland) Nucleo LK432KC board. The node measurements are packeted in a proper JSON and sent via low-energy Bluetooth to a Raspberry Pi (Galles, UK) 3B+ Model. A Wi-Fi router shares the WAN connectivity services with the Raspberry Pi, allowing the data to reach the MONGO DB database server for storage.

Four nodes, named AQ6, AQ8, AQ11, and AQ12, have been deployed against a mobile regulatory air quality monitoring station for a two-months-long winter co-location campaign completed in the city of Portici (Naples, Italy) from 2 January to 2 March 2020. During the campaign, the AQ8 sensor broke, and therefore it will not be considered.

This research study is focused only on nitrogen dioxide NO_2_, which is considered one of the most dangerous pollutants, meaning that the value of the NO_2_ concentration is our target variable ***Y***. In fact, the experimental data stored on the server were averaged on an hourly basis and used to calibrate the sensor nodes with an ad hoc multilinear regression model, characterized by six input features ***X***: relative humidity (RH), temperature (Temp), AE and WE signals of NO_2_ sensor, AE and WE signals of O_3_ sensor [18].

### 2.2. Concept Drift Detection Trigger

Let (***X***, ***Y***) be couples of input-target samples and $P_{t}\left(X,Y\right)$ be its joint probability distribution at the instant time *t*; a concept drift results if $P_{t}\left(X,Y\right)\;\neq \;P_{t+1}\left(X,Y\right)$. This formula can be separated by means of Bayes’ law in the following relation:

(1) $$P_{t}\left(X\right)P_{t}\left(Y|X\right)\;\neq \;P_{t+1}\left(X\right)P_{t+1}\left(Y|X\right)$$

A spatial or temporal change in the characteristics of the features X, but also a change in the relationship linking the inputs to the target variable, is the cause of the decline in the performance of the model used. Therefore, the greater the difference, the greater the error that has been committed. It becomes imperative to be able to identify when a similar event is occurring. Statistical tools are one of the most widely used and well-established methods for accomplishing these tasks. A comprehensive guide to the concept drift detection algorithms is available in [10].

An immediate and simple implementation of a concept drift detection block can be implemented (both on the node hardware and on the back-end server-side software) using the two-sample Kolmogorov–Smirnov test (TSKS test), which is able to evaluate whether two samples come from the same distribution without making any assumption regarding the type of distribution. In the case of low-cost air quality sensors calibrated using ML techniques, the TSKS test can be used to determine whether both the distributions of the input features X and the distributions of the output variable Y during the training phase remain the same also throughout the test phase. Of course, concept drift has occurred, and a recalibration request is triggered if the distributions turn out to be different. Since X-values are always available during the LCAQMS operational scenario, it is possible to detect concept drift even without a reference instrument by comparing the X-distributions obtained during co-location with those obtained during the operational phase (test phase). The same Is true for the target variable. The value in co-location is compared with the value predicted by the model in the test phase. Furthermore, if it is possible to access reference station data from a remote calibration perspective, the same approach is obvious for the output variable. According to the heuristic rule described in [19], a recalibration request is only triggered when the maximum difference between the cumulative density functions of the training and test distributions of the temperature and/or NO_2_ concentration exceeds the threshold of 0.3.

**Figure 1:**
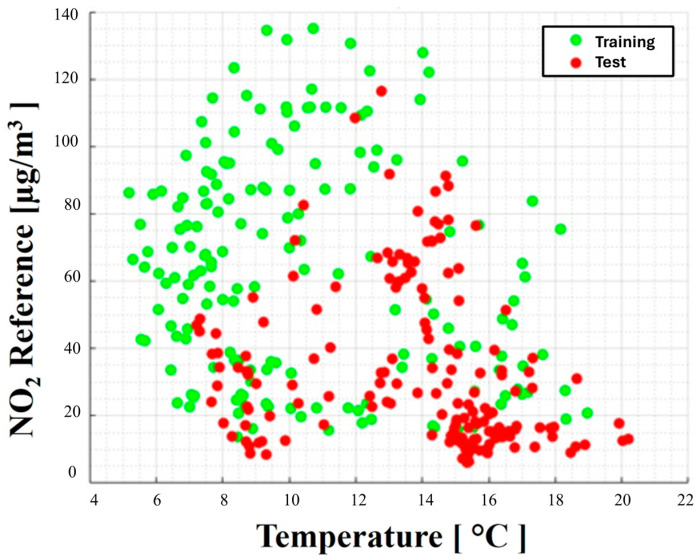
Evidence of concept drift highlighted on the co-location samples of the target and input variables.

By applying this approach to the experimental data collected, divided into eight batches (T1 to T8), each one week long, the presence of two concept drifts has been detected. The first is just before the end of the T4 slot time, while the second drift event happens in T8, but it is not considered in such analysis. A graphic representation of the concept drift existing in the dataset under analysis is shown in Figure 1. The green dots represent the trend of the “concept” that characterizes the target variable and an input variable (temperature, in this case) during the co-location period and, therefore, during the training process. The red dots represent the trend of the same variables after the concept drift detection; therefore, the “new concept” characterizes the test set after only one month.

### 2.3. Calibration Model Update Data Selection

To tackle the problem of concept drift handling, data were analyzed to find those containing the useful information to update the calibration model.

**Figure 2:**
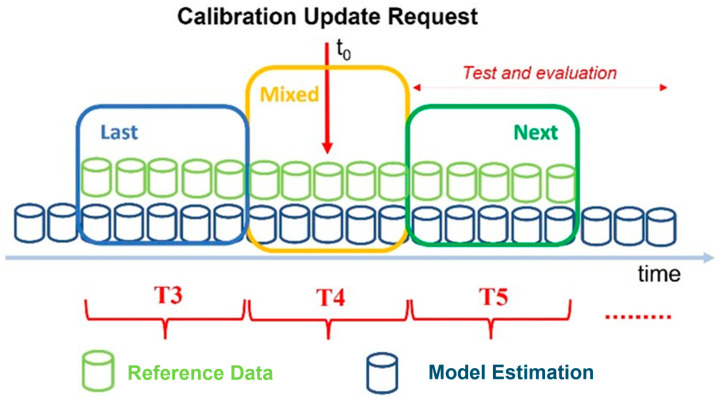
Data selection for calibration update when a recalibration request arrives from the concept drift detector.

Let *t*_0_ be the time at which the concept drift detector block implemented in the air quality monitoring network sends an alarm signal to the network administrator, meaning that the current calibration model is obsolete and needs to be updated. Now the first question is to understand which data should be used for the update. Three possible reference datasets can be used within this scope: the dataset preceding the concept drift alert, called “Last” and identified with the T3 time slot (Figure 2); the dataset following the concept drift alert, called “Next” (T5 in Figure 2); or part of both called “Mixed” that contains the time instant *t*_0_ and is reported as the T4 time slot (Figure 2) [20].

Our attention will focus on trying to restore the compliant data quality. The following tests are fulfilled by updating the model using on-hand reference data (in the proper time windows) and then evaluating the performance of the new model in T5–T6–T7.

### 2.4. Calibration Update without Reference Data

Bringing back the data quality at the regulatory level after the detection of a concept drift event remains the final goal of our study. This means the mitigation of the effects related to the concept drift. To make the most out of the information content of the co-location data, two new calibration models beyond the ad hoc one were extracted from it. The following techniques were explored: the general (also named global or, less often, universal) calibration model, and the importance weighting calibration model.

#### 2.4.1. General Calibration Model

**Figure 3:**
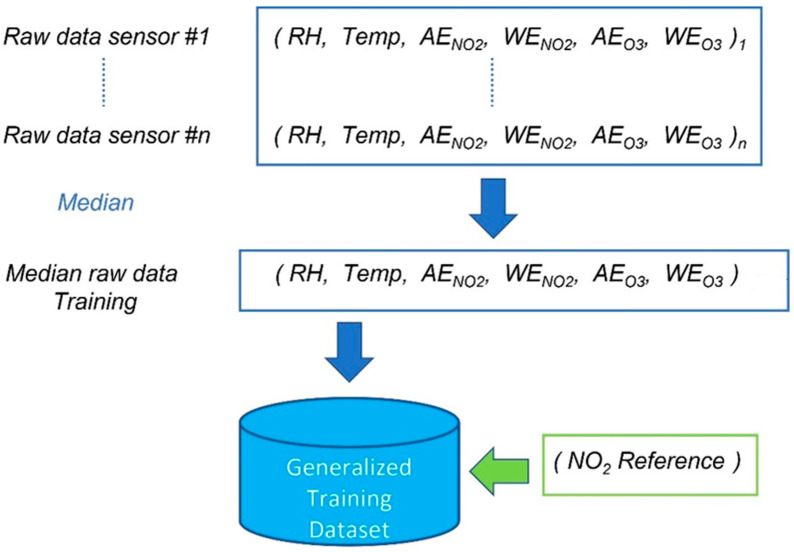
Procedure for creating the training set of the general calibration model.

This methodology has been introduced in recent years as an attempt to reduce the calibration costs [21,22]. It consists of identifying and applying a general calibration model to all the nodes involved in the network, thus avoiding the need for additional ad hoc calibrations. As known, the raw output of most electrochemical gas sensors consists of the voltage (mV) measured at the working and auxiliary electrodes that is representative of the measured gas concentration. Let us consider *n* co-located sensors, and then the median between these *n* values is calculated. The same procedure is applied for temperature and humidity values. The set of medians of all the single quantities (input variables) involved in model creation accounts for the training set which the global model is trained on. Such a procedure is depicted in Figure 3.

Such an approach can incorporate the inherent variability of each sensor into a single model, as previously investigated to contrast the effects of concept drift on metal oxide (MOX) gas sensors [23]. The same approach was applied to the NO_2_ electrochemical gas sensors in our case study.

#### 2.4.2. Importance Weighting Calibration Model

The idea behind this procedure is to “weigh” the test set samples in order to “match” the distribution used during the training phase. Once the weights are obtained, these will be applied in the training process, obtaining a new calibration model [24].

**Figure 4:**
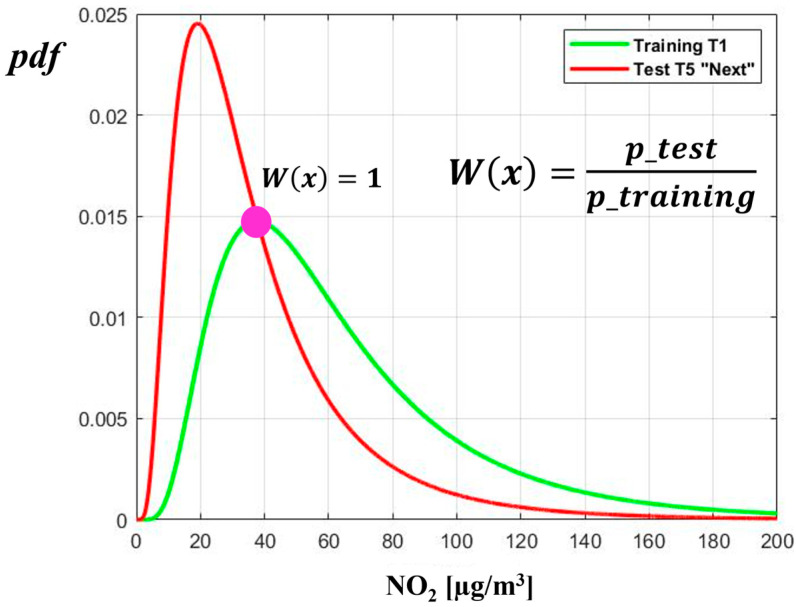
How the weights are used in the fitting process in MATLB fitlm (*x*, *y*, ’Weight’, w) function.

The importance of a sample (i.e., the “weight”) is calculated as the ratio between the probability density functions (*pdf*) of the test and training set (Figure 4). If such a ratio *w*(*x*) is equal to 1, this means that the sample has the same degree of importance in both the test and the training set, while if *w*(*x*) > 1, the considered sample is more important in describing the test set rather than the training set.

In the present analysis, the application of the importance of the weighting calibration model was limited to the target variable only, and the weights were obtained using the ad hoc model prediction of the time slot T4 (the last useful time slot before the effects of the concept drift would become disruptive).

#### 2.4.3. Stacking Ensemble Calibration Model

Recently, the stacking ensemble technique has been successfully applied in domain adaptation under concept drift due to its ability to reduce the deviation and variance in neural networks, thus producing robust predictions. Such a strategy has also already been applied in air quality sensor networks [25,26].

**Figure 5:**
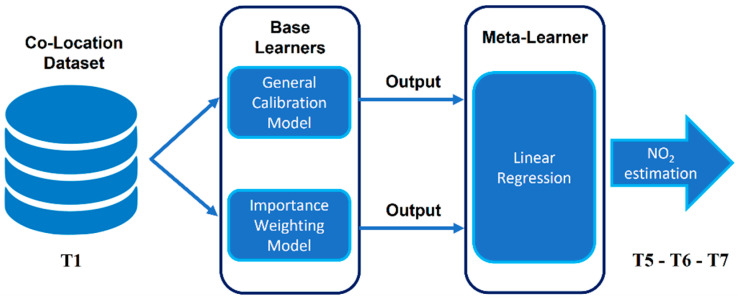
The proposed stacking ensemble architecture.

Stacking ensemble consists of combining the outputs of several models produced by different algorithms (generally called *base learners* or also *weak models*) in order to increase the total accuracy and increase generalization. The estimations of the basic learners are combined in a second level, called meta-learner, which, in the case of regression problems, can be a simple linear regression. In our case study, instead of implementing additional algorithms such as base learners, we exploited the estimations of nitrogen dioxide resulting from the general calibration model and the importance weighting calibration model. Figure 5 describes the proposed architectural scheme in detail. The reference data (labels) for the meta-learner training phase will be requested to the air quality network in the T4 time slot. Such an approach is known as remote calibration, as described in the Introduction, and in our case study, its usage avoids new co-locations.

#### 2.4.4. Performance Evaluation

According to the European Directive [27], if a NO_2_ low-cost sensor is used in an air quality network for indicative measurements, it should present a relative expanded uncertainty (REU) below 25%. In this analysis, we labeled this positive operational scenario with “PASS”, while “FAIL” identified REU > 25%. The REU and the REU plots, reported in the Supplementary Materials, were computed with an appropriate MATLAB script on the basis of the formulas and the methodology described in [19,28]. In our analysis, another main parameter that was taken into account was the pollutant concentration value, at which the REU graph intercepts the data quality objective (DQO) threshold line at 25%. Obviously, the following choice between different models would be oriented towards the model that shows the lowest possible REU, which is translated into the model’s better capability to mitigate the effects of the concept drift.

## 3. Results and Discussion

To improve the readability of this section, the reader can refer directly to the following tables, which summarize the outcomes of the REU plots, which are detailed for completeness in the Supplementary Material.

### 3.1. Results with Reference Data

It would be obvious to come to the conclusion that the “Next” approach is to be preferred over the others because it is after the alert that the new operating conditions fully manifest themselves, independently, from the metrics used for the assessment.

However, in order to learn the new operating domain, it is necessary to wait for the necessary time to acquire a certain number of samples to train the new model. This means continuing, in the meantime, to invalidate the data released by the node (which would be a PASS level of REU up to T4 and FAIL from T5 onward). An important drawback is represented by the delayed time needed to obtain reference data before having a running updated model. It could be possible to wait for T5 reference data and then embed the new concept into the new updated model. However, such a strategy, even if allowing for good performances in T6 and T7, would lead to a loss of continuity in the data quality in T5. The goal, instead, is to achieve a continuity in data quality by wishing the REU in T5–T6–T7 to be equal to PASS. Therefore, it would be helpful if the “Last” and/or “Mixed” approaches presented acceptable REU values.

Table 1 and Table 2 summarize the results obtained from the REU plots in the “Mixed” and “Last” scenarios and emphasize their ability to mitigate the concept drift effects and lead to more accurate and precise NO_2_ estimations. Both tables show, for each time slot and for each device, the assigned REU labels (PASS, FAIL) as well as the value corresponding to the intersection of the pollutant concentration value with the data quality objective (DQO) threshold line at 25%, as disposed by the European Directive [27]. The missing values in the tables are related to the REU plot, failing to reach the minimum threshold of 25%.

Observing the REU plots from T5 to T7 for the AQ12 device in the “Mixed” approach, it can be seen that the REU drops below 25% from 55 µg/m^3^ onwards in T5 and T6 (Figures S1.1 and S1.2), while it drops from 51 µg/m^3^ in T7 (Figure S1.3). Surely such a situation is to be preferred since it allows for the continuity of data quality, extending the hoped PASS REU value for 3 more weeks. Regarding the AQ11 device, the “Mixed” approach worked similarly to AQ12, but the inherent variability of the device negatively affected the model performance, meaning it failed to repair the effects of the concept drift. In fact, it can be seen that the intersections with the DQO threshold line at 25% are higher, at 60 µg/m^3^ in T5 (Figure S1.4) and 80 µg/m^3^ in T6 and T7 (Figures S1.5 and S1.6), respectively. On the other hand, in the “Last” approach, AQ11 exhibits good performances only in T5 (Figure S1.7), while it does not reach the desired REU levels in T6 and T7 (Figures S1.8 and S1.9). This means that the intrinsic variability of AQ11 and the operating conditions in T3 used for the fitting of the new model are too different from those present after the concept drift. The AQ6 device in the “Mixed” approach presents a FAIL in T5, while in T6 and T7, a PASS is reached at 65 and 70 µg/m^3^, respectively (Figures S1.10–S1.12). The “Last” scenario for AQ6 does not work, except for in T6, but at a higher pollution level of 80 µg/m^3^ (Figures S1.13–S1.15). The outcomes for the AQ12 device in the “Last” scenario are missing due to data transmission issues.

From the analysis carried out, it can be concluded that all in all, if there is a possibility of drawing on the data of a regulatory reference station, it is possible to update the calibration model after the trigger signal using the “*Mixed*” approach, i.e., selecting part of the reference data before the concept drift alert and remaining data after it.

### 3.2. Results without Reference Data: General Calibration Model

Proceeding similarly to the previous case, it is essential to evaluate the REU to understand if the general calibration model is able to positively mitigate the effects of the concept drift. Table 3 details the obtained results, while the REU plots in the Supplementary Materials are also shown in comparison with the ad hoc calibration model.

Applying the general calibration model to the AQ6 node, the REU plot drops below 25% at 45 µg/m^3^, suggesting that the global calibration model is efficient in mitigating the concept drift consequences in the T5 time slot (Figure S2.1). This value shifts at higher concentration levels in T6 and T7, specifically at 63 µg/m^3^ and 80 µg/m^3^, respectively (Figures S2.2 and S2.3). Although the REU does not fall within a considerable range of values below 25% in T6 and T7, it must be observed that such a situation presents better results than the ad hoc model. Therefore, a first applicative scenario would be to use the global model for AQ6 from T5 onwards, instead of continuing with the ad hoc one. For the AQ12 device, the general calibration model matches the ad hoc model performance (Figures S2.4–S2.6). The same result has been obtained for particulate matter sensors in terms of the mean absolute error (MAE) in [29]. The AQ11 intrinsic node variability makes this instrument too different from the others; therefore, a general model built in this way is unable to work properly for our purposes. Indeed, its performance is too far also from the performance of the ad hoc model; thus, we chose not to report the REU plots. Future research projects could investigate this aspect and evaluate whether it is better to build more global models among similar devices by means of clustering [30].

### 3.3. Results without Reference Data: Importance Weighting Calibration Model

As can be seen in Table 4, re-weighing the target variable in T4 produces a considerable improvement in the performance of the AQ11 device. In fact, looking at the REU plot of AQ11 in T5, the intersection with the 25% value was observed at 40 µg/m^3^ (Figure S3.4), while in T6 and T7, it did not quite reach the DQOs, although the importance weighting calibration model performs slightly better than the ad hoc one (Figures S3.5 and S3.6). As far as the AQ6 and AQ12 devices, the REU plots equaled those of the ad hoc model in some time slots, while they were worse in others (Figures S3.1–S3.3 and S3.7–S3.9).

Summarizing the application of the importance weighting calibration model brings the AQ11 device in T5 back to the allowed REU values.

### 3.4. Stacking Ensemble Calibration Model Results

Table 5 shows the summary of the REU values for the stacking ensemble calibration model. The inference to be drawn comparing the outcomes of Table 5 with those of Table 1 is that the application of the stacking ensemble technique lowers the value at which the REU reaches 25%, making the mitigation of the concept drift effects more robust in all the analyzed cases. In principle, the use of better-performing base learner algorithms could contribute to further error reductions. The proposed solution, although not yet optimal, constitutes a good compromise, since it guarantees the continuity of the data’s quality even after the detection of concept drifts. Furthermore, operating within an air quality network, the implementation of the proposed stacking ensemble approach requires only the reference data in T4, meaning that any other re-co-location could be avoided in a remote calibration approach.

## 4. Conclusions

In this study, we attempted to achieve the data quality objectives in the measurements of environmental pollutants through machine-learning-calibrated low-cost gas sensors. Through our results, we hope to accelerate the spread of this technology in smart city applications. The core of our work is a concept drift adaptation in the context of air quality monitoring networks but by exploring the possibility of using the whole useful information content of the data collected in the course of the co-location.

Another noteworthy aspect relates to the choice of calibration models used. With the general calibration model, we wanted to incorporate the intrinsic variability of each sensor into a single calibration model. On the other hand, with the importance weighting calibration model, we tried to reduce the prediction error by matching the distributions between training and test using accurate weights. Unfortunately, the general calibration model and the importance weighting calibration model show promising results only for some devices and only in the one time slot in this specific dataset. On the contrary, their use in a stacking ensemble architecture succeeds in mitigating the concept drift effects by allowing an extension of the calibration robustness both for all devices and in all time slots after concept drift. For example, by turning off the ad hoc model and turning on the stacking ensemble model, the node is able to output reliable data.

Even though the proposed methodology offers encouraging results, future research is encouraged to test other models in the first layer of the stacking ensemble to increase performance but also to test the assessment and validation of the methodology using wider datasets with a greater number of devices.

Finally, an important aspect to remark is the feasibility of the implementation on the backend side of the platform of an air quality network of the proposed approach. This might deliver a continuous calibration service. Recently, indeed the issue of evaluating and monitoring machine learning models from validation to production has come to the fore. Therefore, numerous start-ups have implemented their own platform and began offering services in many application areas [31]. The low-cost air quality monitoring network could be one of such applications.

## Figures and Tables

**Table 1:** Summary of REU results for the “Mixed” scenario.

	T5		T6		T7	
	REU	Value [µg/m^3^]	REU	Value [µg/m^3^]	REU	Value [µg/m^3^]
**AQ6**	FAIL	-	PASS	65	PASS	70
**AQ11**	PASS	60	PASS	80	PASS	80
**AQ12**	PASS	55	PASS	55	PASS	51

**Table 2:** Summary of REU results for the “Last” scenario.

	T5		T6		T7	
	REU	Value [µg/m^3^]	REU	Value [µg/m^3^]	REU	Value [µg/m^3^]
**AQ6**	FAIL	-	PASS	80	FAIL	-
**AQ11**	PASS	55	FAIL	-	FAIL	-

**Table 3:** Summary of REU results for the general calibration model.

	T5		T6		7	
	REU	Value [µg/m^3^]	REU	Value [µg/m^3^]	REU	Value [µg/m^3^]
**AQ6**	PASS	45	PASS	63	PASS	80
**AQ11**	FAIL	-	FAIL	-	FAIL	-
**AQ12**	FAIL	-	FAIL	-	FAIL	-

**Table 4:** Summary of REU results for the importance weighting calibration model.

	T5		T6		T7	
	REU	Value [µg/m^3^]	REU	Value [µg/m^3^]	REU	Value [µg/m^3^]
**AQ6**	FAIL	-	FAIL	-	FAIL	-
**AQ11**	PASS	40	FAIL	-	FAIL	-
**AQ12**	FAIL	-	FAIL	-	FAIL	-

**Table 5:** Summary of REU results for the stacking ensemble calibration model.

	T5		T6		T7	
	REU	Value [µg/m^3^]	REU	Value [µg/m^3^]	REU	Value [µg/m^3^]
**AQ6**	PASS	44	PASS	56	PASS	65
**AQ11**	PASS	44	PASS	70	PASS	76
**AQ12**	PASS	51	PASS	54	PASS	45

## Data Availability

Supporting data are currently available on request.

## Supplementary Materials

The following supporting information can be downloaded at: https://www.mdpi.com/article/10.3390/s24092786/s1, Figures S1.1–S1.15: REU plots of reference data selection for calibration update; Figures S2.1–S2.6: REU plots of general calibration model; Figures S3.1–S3.9: REU plots of importance weighting calibration model; Figures S4.1–S4.9: REU plots of stacking ensemble calibration model.
