# Supplementary material for: Concept Drift Mitigation in Low-Cost Air Quality Monitoring Networks
Source: Sensors (Basel). 2024 Apr 27;24(9):2786. doi: 10.3390/s24092786 (PMC11086340; doi:10.3390/s24092786)
Supplement: Supplementary file 1 [file sensors-24-02786-s001.zip › sensors-2925793-supplementary.pdf]

Supplementary Materials

Concept Drift Mitigation in Low-Cost Air Quality Monitoring Networks

Gerardo D'Elia [1,2,*], Matteo Ferro [3], Paolo Sommella [2], Sergio Ferlito [1], Saverio De Vito [1] and Girolamo Di Francia [1]

[1] TERIN-FSD-SAFS Laboratory, ENEA CR-Portici, P. le E. Fermi 1, 80055 Portici, Italy
[2] Department of Industrial Engineering (DIIn), University of Salerno, Via Giovanni Paolo II, 132, 84084 Fisciano, Italy
[3] Hippocratica Imaging S.r.l., Via Giulio Pastore, 32, 84131 Salerno, Italy
* Correspondence: gerardo.delia@eneS.it; Tel.: +39 081 7723 616

S.1 REU plots of reference data selection for calibration update

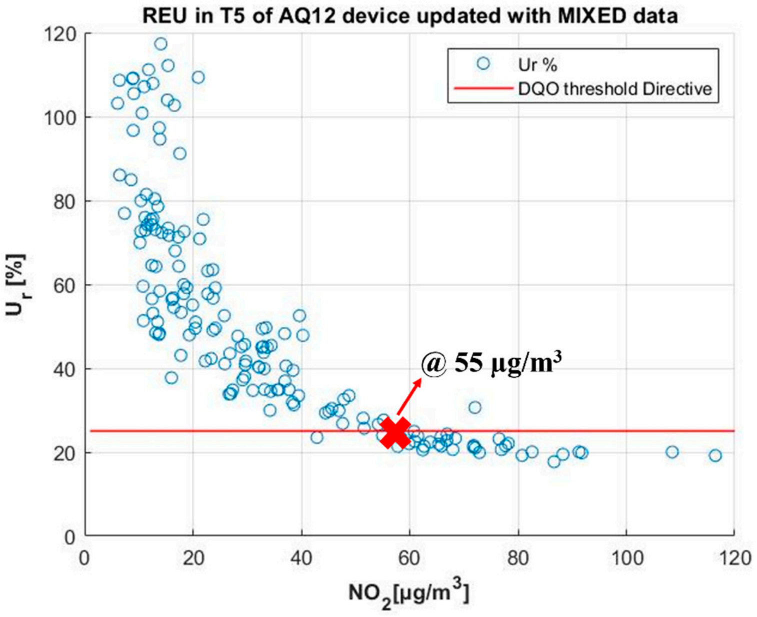

Figure S.1. 1 *Plot of Relative Expanded Uncertainties in T5 when AQ12 is re-calibrated with data of T4 (Mixed scenario).*

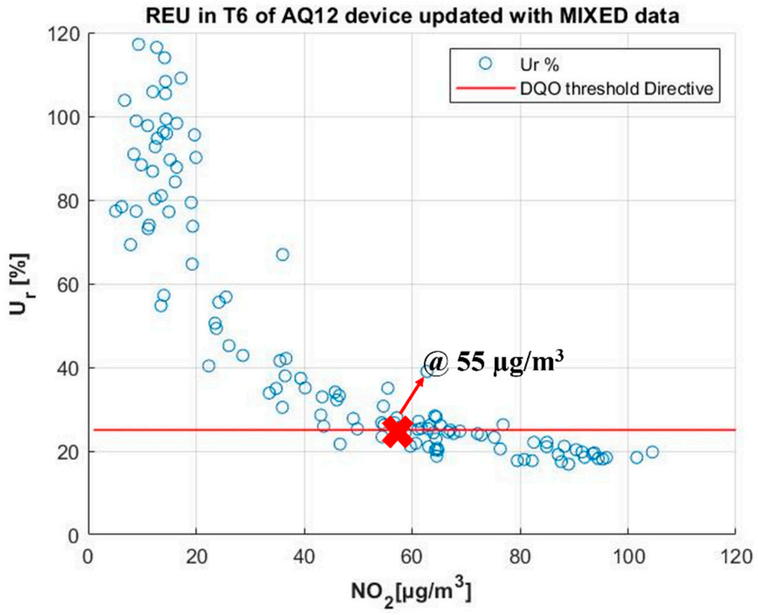

Figure S.1. 2 *Plot of Relative Expanded Uncertainties in T6 when AQ12 is re-calibrated with data of T4 (Mixed scenario).*

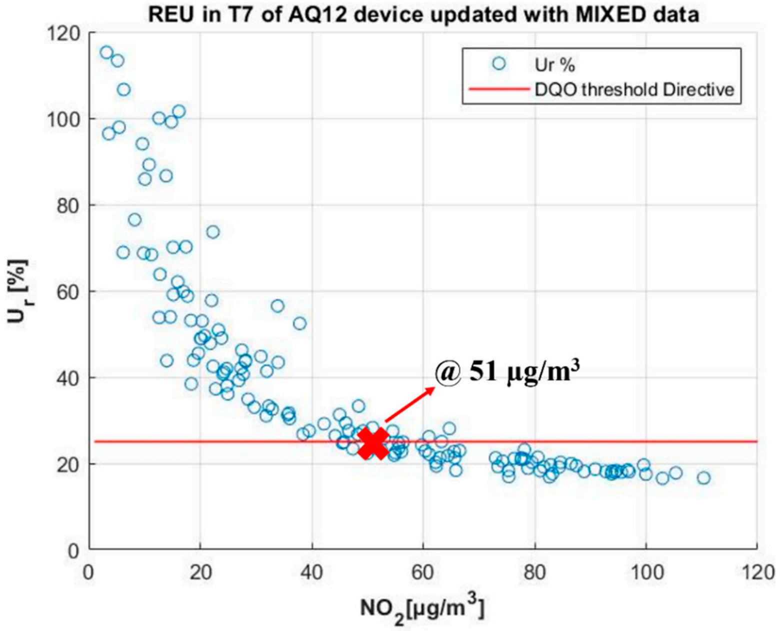

Figure S.1. 3 *Plot of Relative Expanded Uncertainties in T7 when AQ12 is re-calibrated with data of T4 (Mixed scenario).*

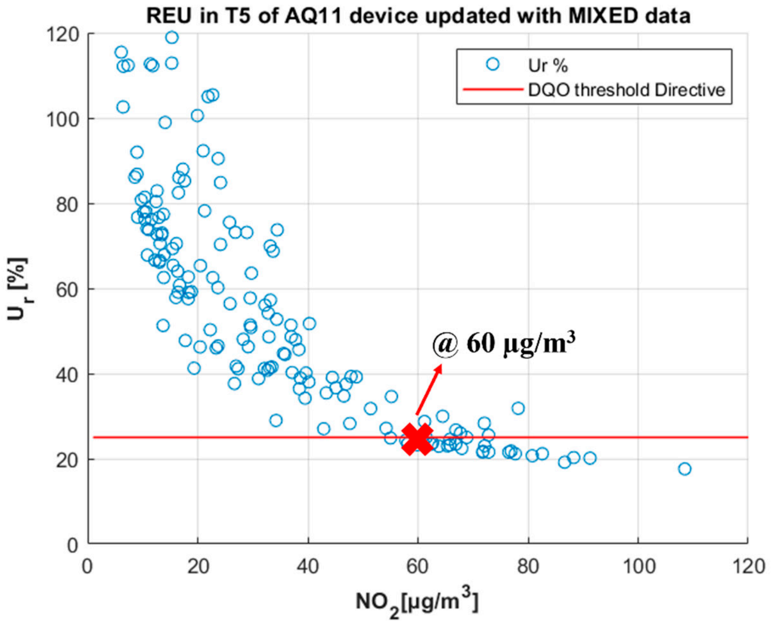

Figure S.1. 4 *Plot of Relative Expanded Uncertainties in T5 when AQ11 is re-calibrated with data of T4 (Mixed scenario).*

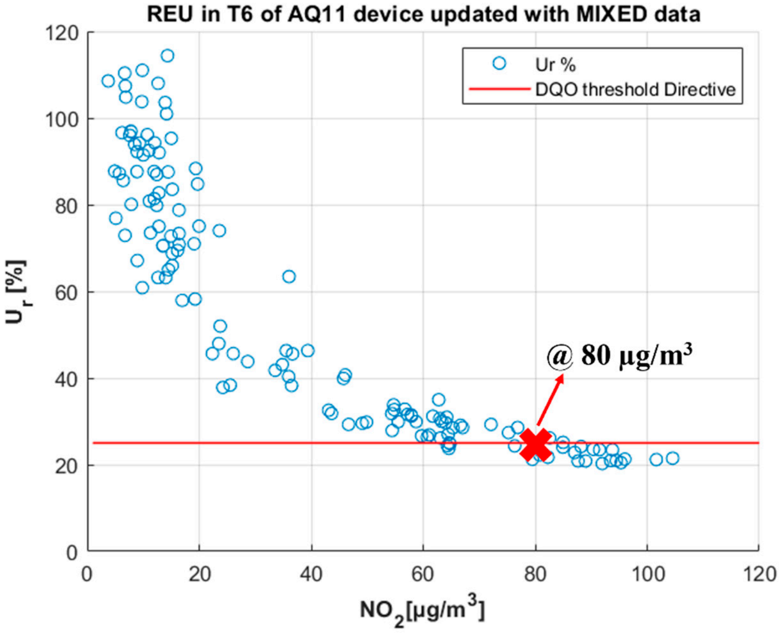

Figure S.1. 5 *Plot of Relative Expanded Uncertainties in T6 when AQ11 is re-calibrated with data of T4 (Mixed scenario).*

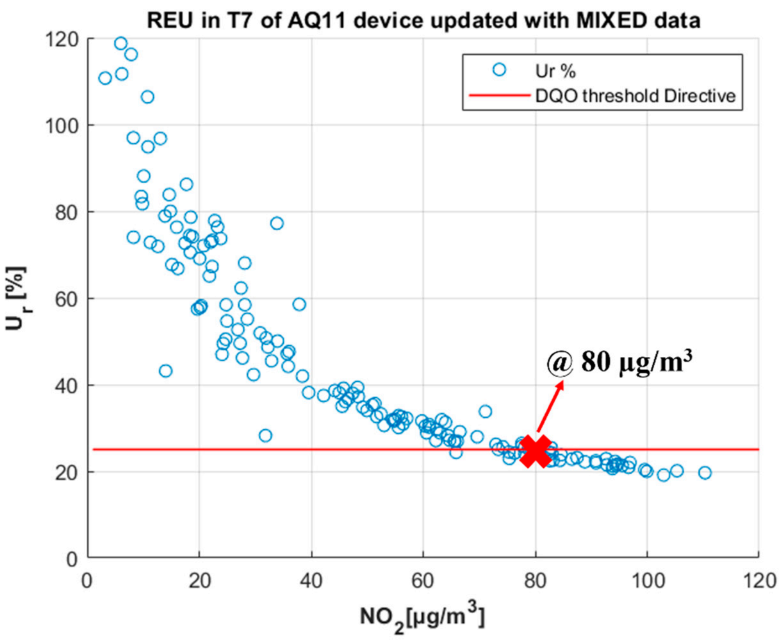

Figure S.1. 6 *Plot of Relative Expanded Uncertainties in T7 when AQ11 is re-calibrated with data of T4 (Mixed scenario).*

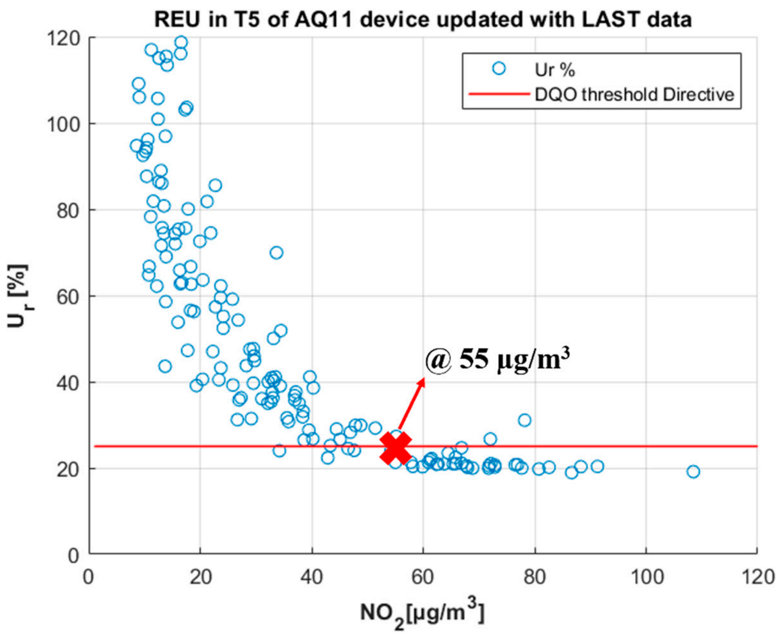

Figure S.1. 7 *Plot of Relative Expanded Uncertainties in T5 when AQ11 is re-calibrated with data of T3 (Last scenario).*

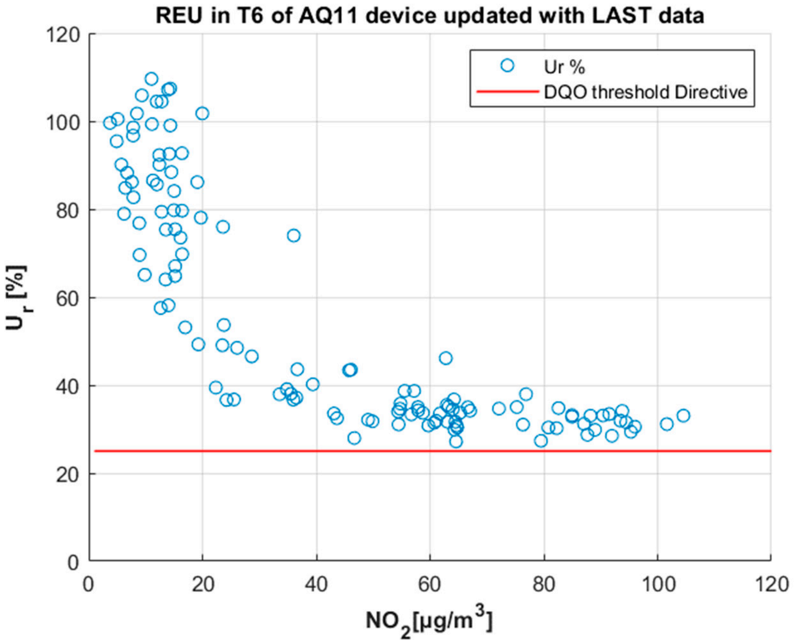

Figure S.1. 8 *Plot of Relative Expanded Uncertainties in T6 when AQ11 is re-calibrated with data of T3 (Last scenario).*

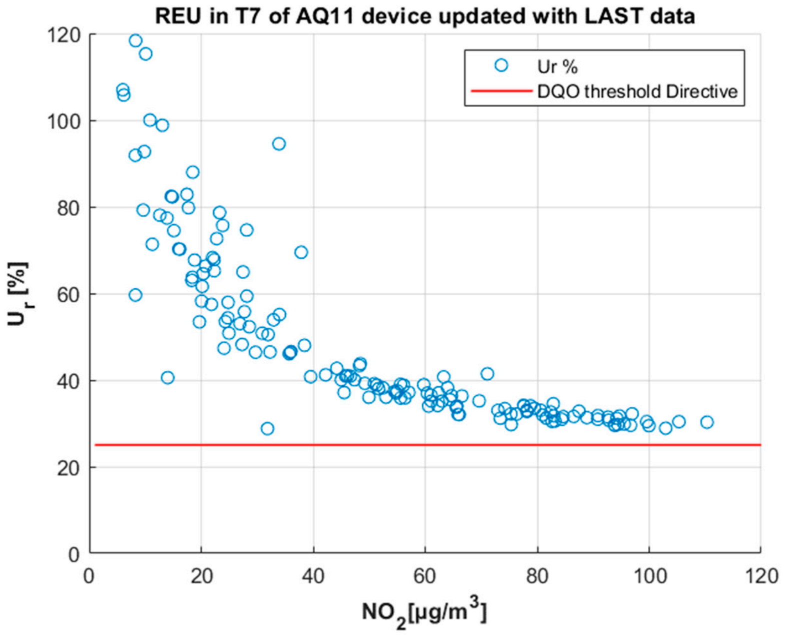

Figure S.1. 9 *Plot of Relative Expanded Uncertainties in T7 when AQ11 is re-calibrated with data of T3 (Last scenario).*

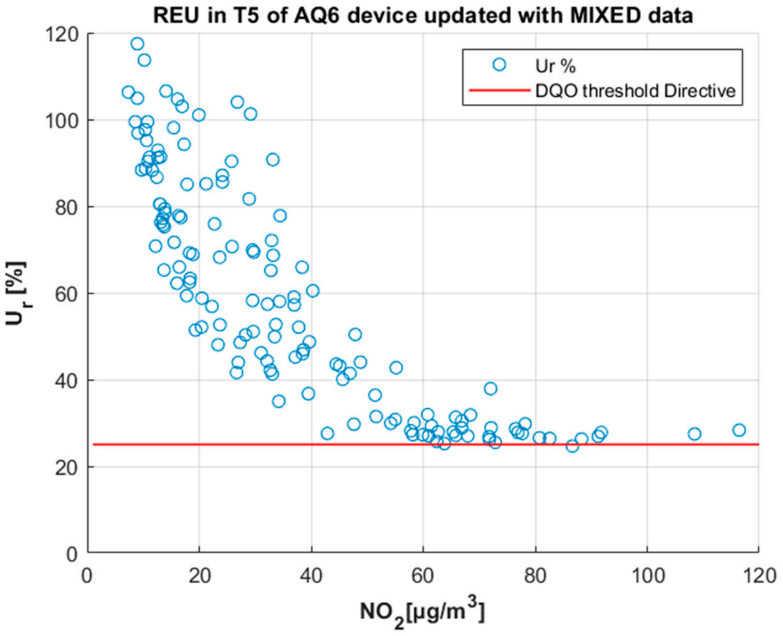

Figure S.1. 10 *Plot of Relative Expanded Uncertainties in T5 when AQ6 is re-calibrated with data of T4 (Mixed scenario).*

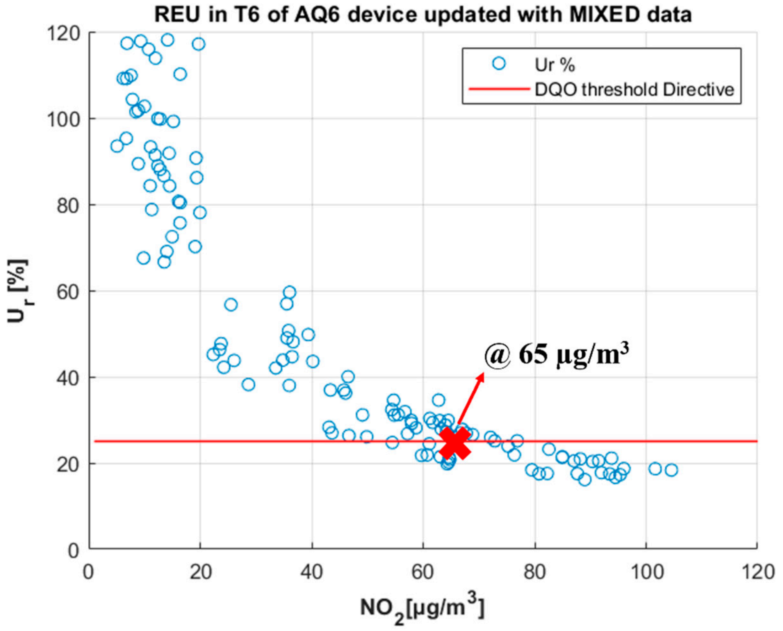

Figure S.1. 11 *Plot of Relative Expanded Uncertainties in T6 when AQ6 is re-calibrated with data of T4 (Mixed scenario).*

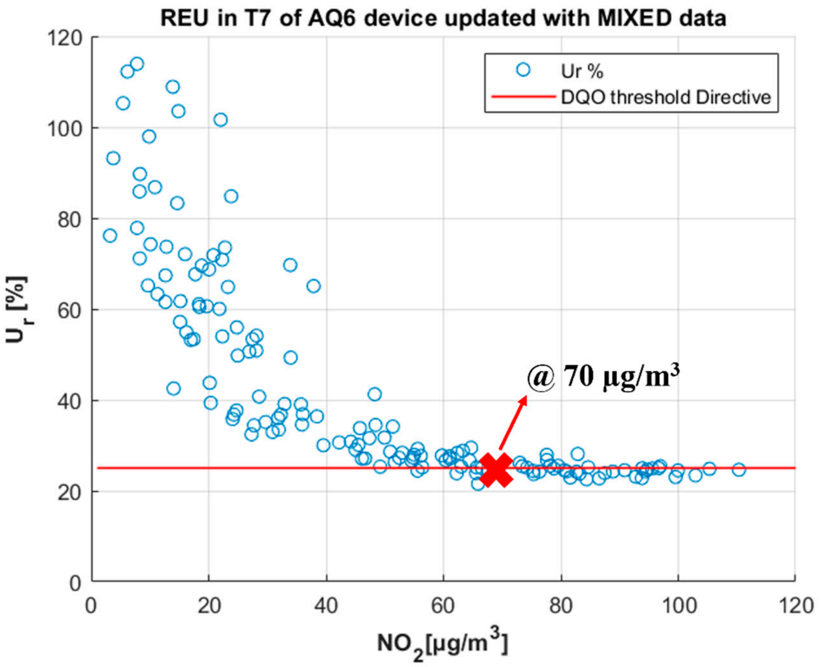

Figure S.1. 12 *Plot of Relative Expanded Uncertainties in T7 when AQ6 is re-calibrated with data of T4 (Mixed scenario).*

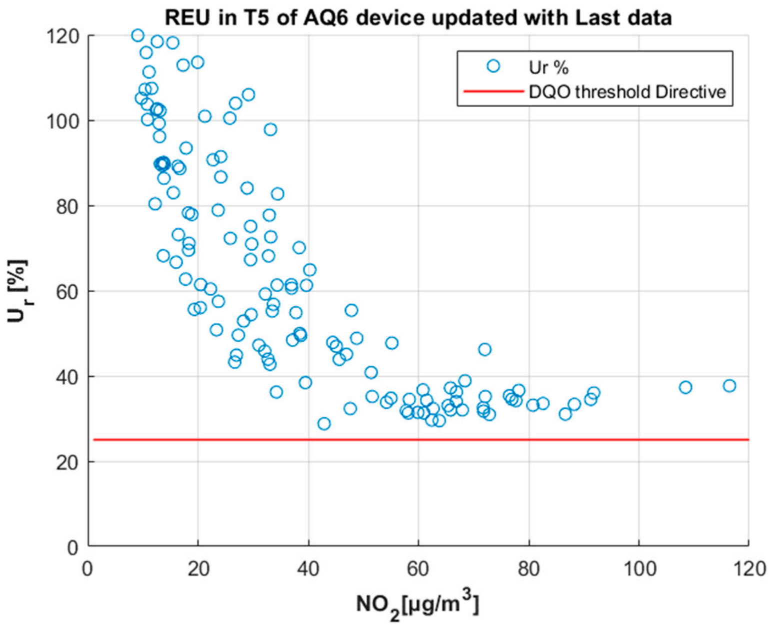

Figure S.1. 13 *Plot of Relative Expanded Uncertainties in T5 when AQ6 is re-calibrated with data of T3 (Last scenario).*

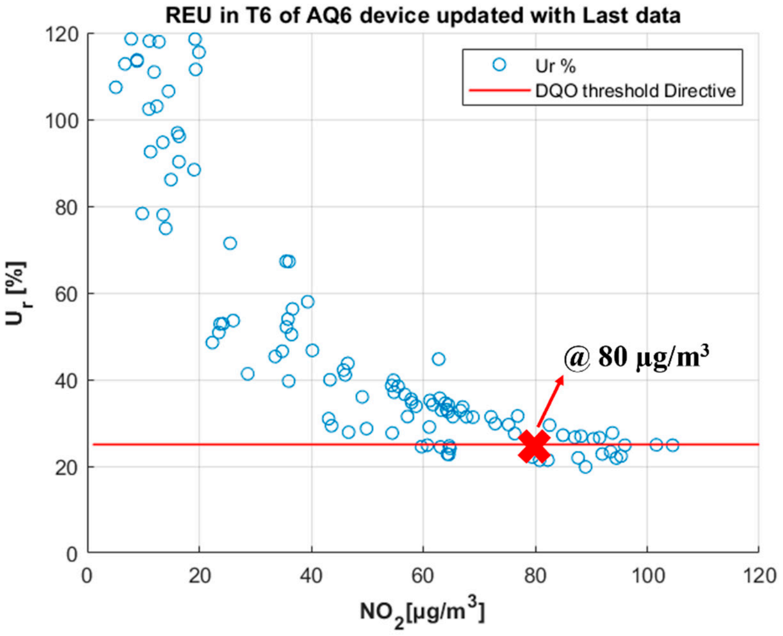

Figure S.1. 14 *Plot of Relative Expanded Uncertainties in T6 when AQ6 is re-calibrated with data of T3 (Last scenario).*

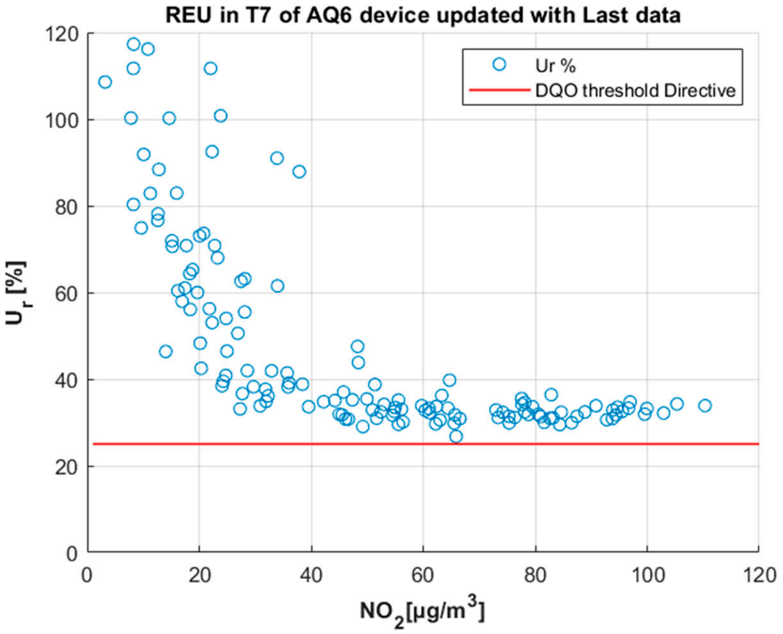

Figure S.1. 15 *Plot of Relative Expanded Uncertainties in T7 when AQ6 is re-calibrated with data of T3 (Last scenario).*

S.2 REU plots of general calibration model

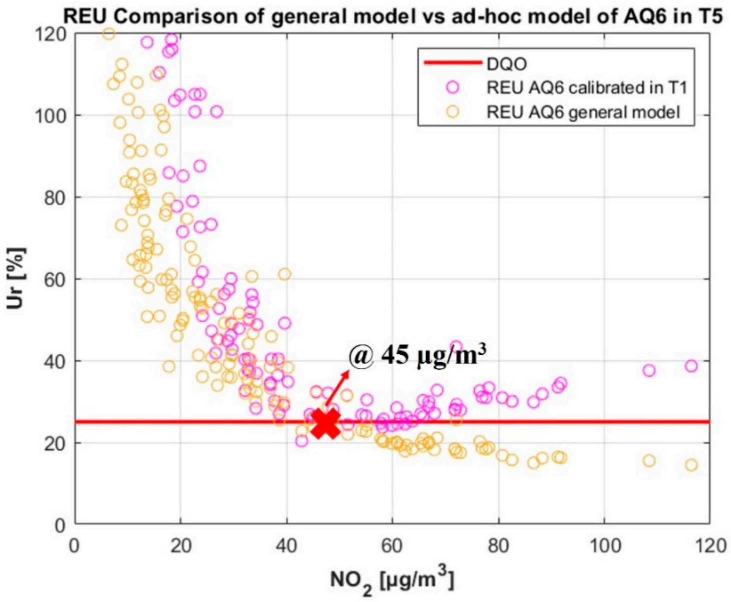

Figure S.2. 1 *Plot of Relative Expanded Uncertainties in T5 when AQ6 is re-calibrated with global calibration model.*

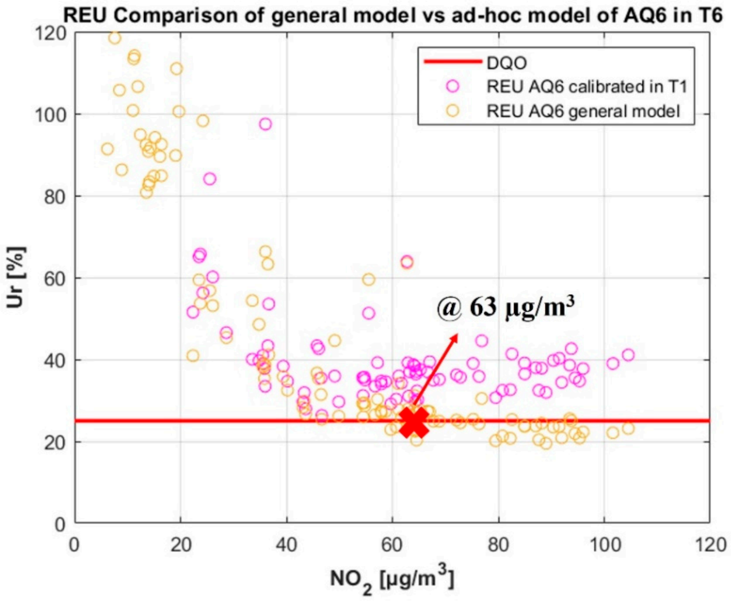

Figure S.2. 2 *Plot of Relative Expanded Uncertainties in T6 when AQ6 is re-calibrated with global calibration model.*

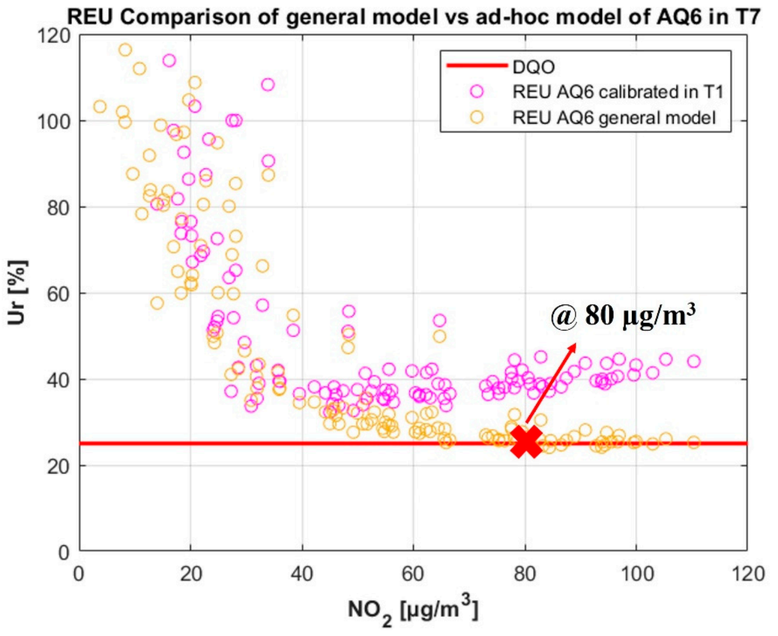

Figure S.2. 3 *Plot of Relative Expanded Uncertainties in T7 when AQ6 is re-calibrated with global calibration model.*

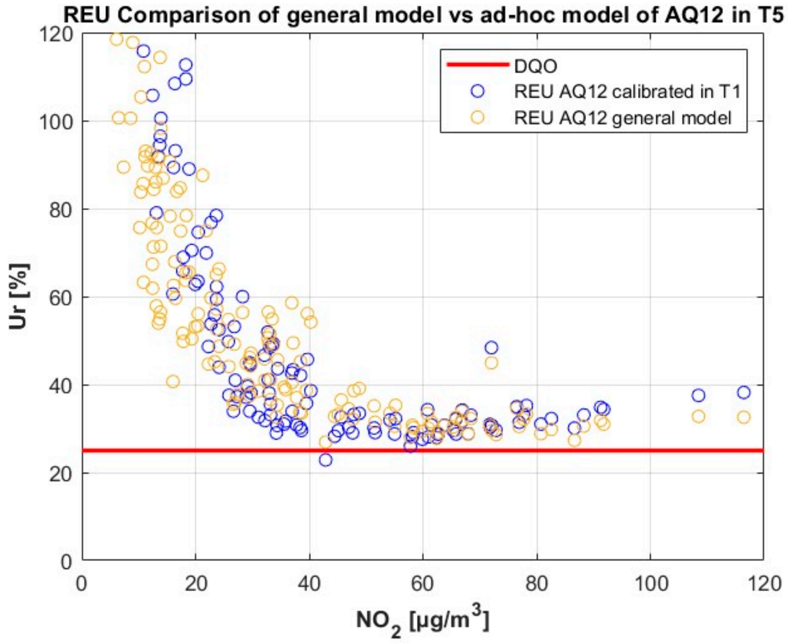

Figure S.2. 4 *Plot of Relative Expanded Uncertainties in T5 when AQ12 is re-calibrated with global calibration model.*

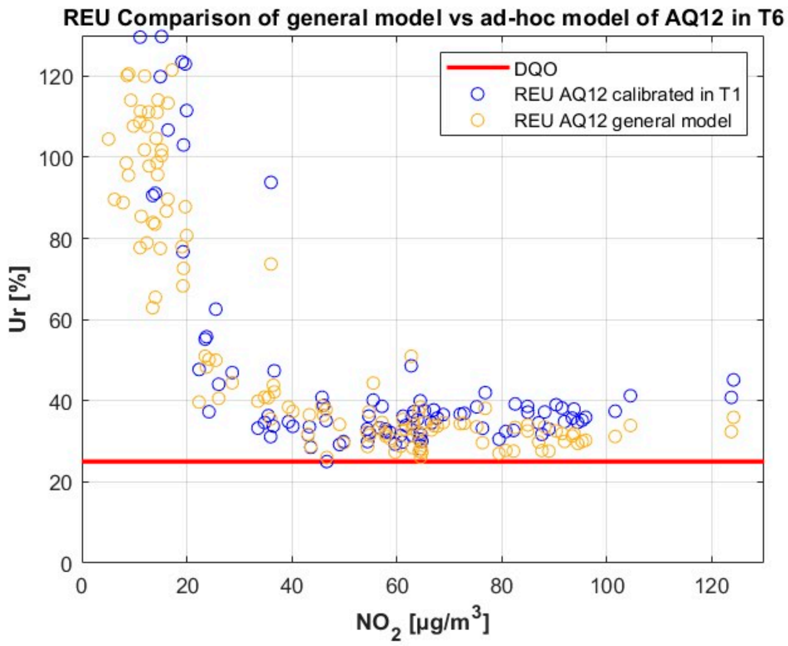

Figure S.2. 5 *Plot of Relative Expanded Uncertainties in T6 when AQ12 is re-calibrated with global calibration model.*

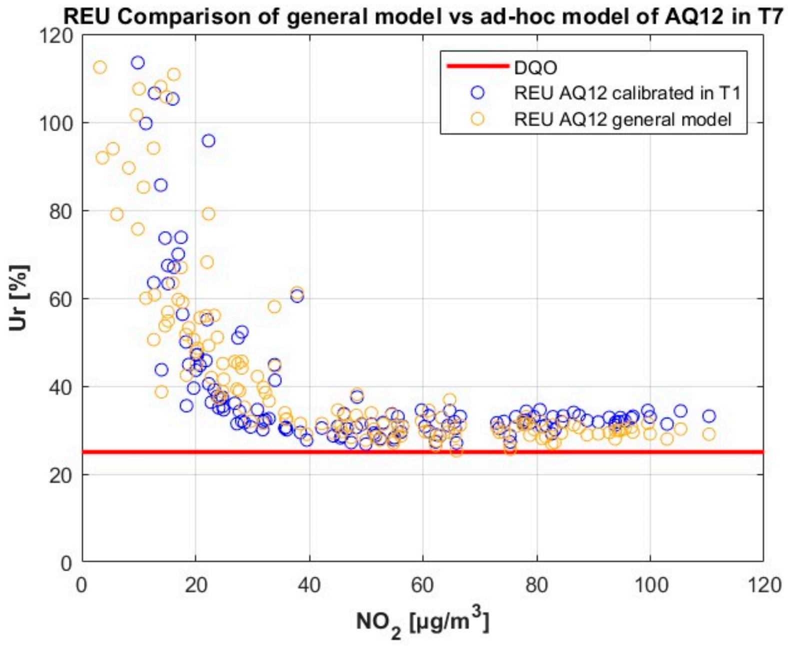

Figure S.2. 6 *Plot of Relative Expanded Uncertainties in T7 when AQ12 is re-calibrated with global calibration model.*

S.3 REU plots of importance weighting calibration model

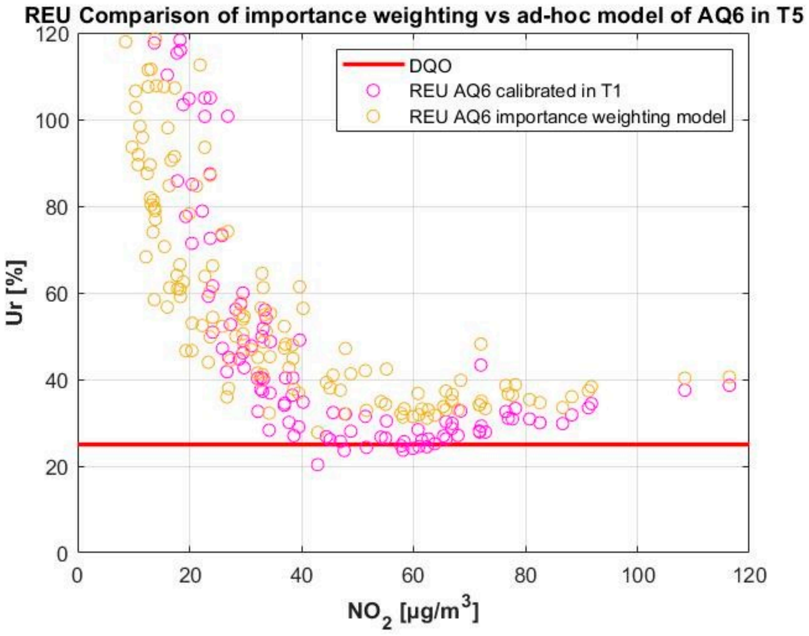

Figure S.3. 1 *Plot of Relative Expanded Uncertainties in T5 when AQ6 is re-calibrated with the importance weighted calibration model.*

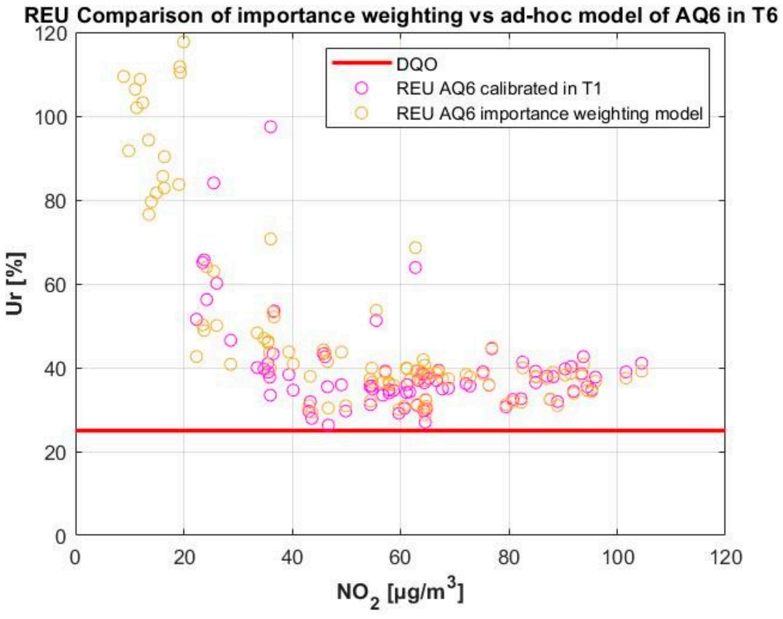

Figure S.3. 2 *Plot of Relative Expanded Uncertainties in T6 when AQ6 is re-calibrated with the importance weighted calibration model.*

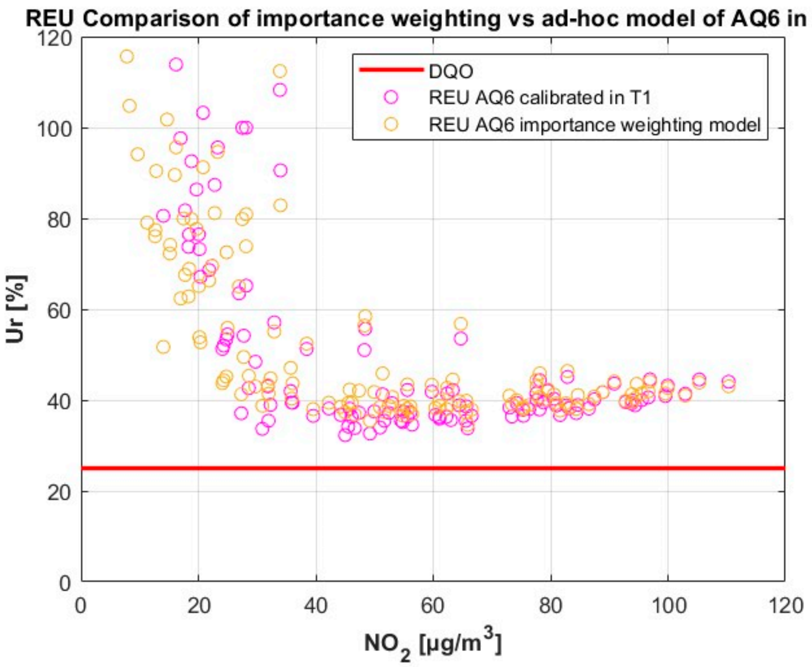

Figure S.3. 3 *Plot of Relative Expanded Uncertainties in T7 when AQ6 is re-calibrated with the importance weighted calibration model.*

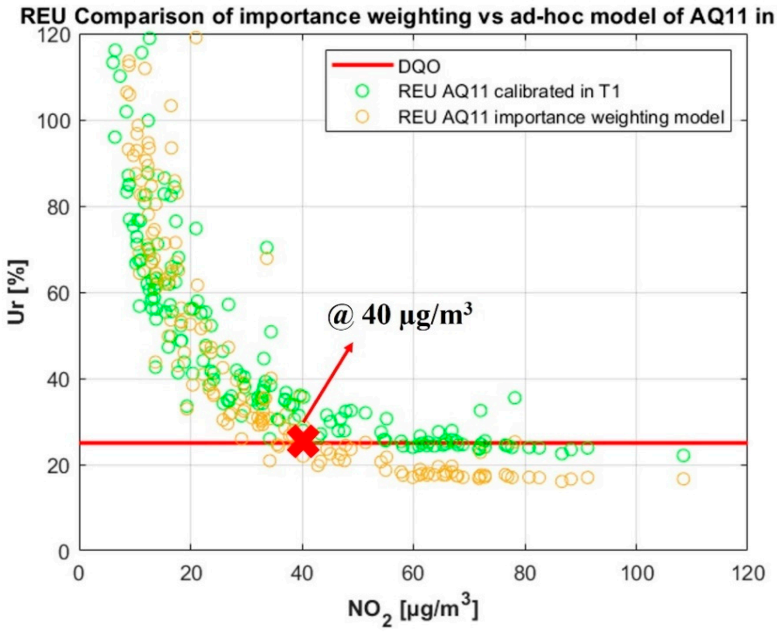

Figure S.3. 4 *Plot of Relative Expanded Uncertainties in T5 when AQ11 is re-calibrated with the importance weighted calibration model.*

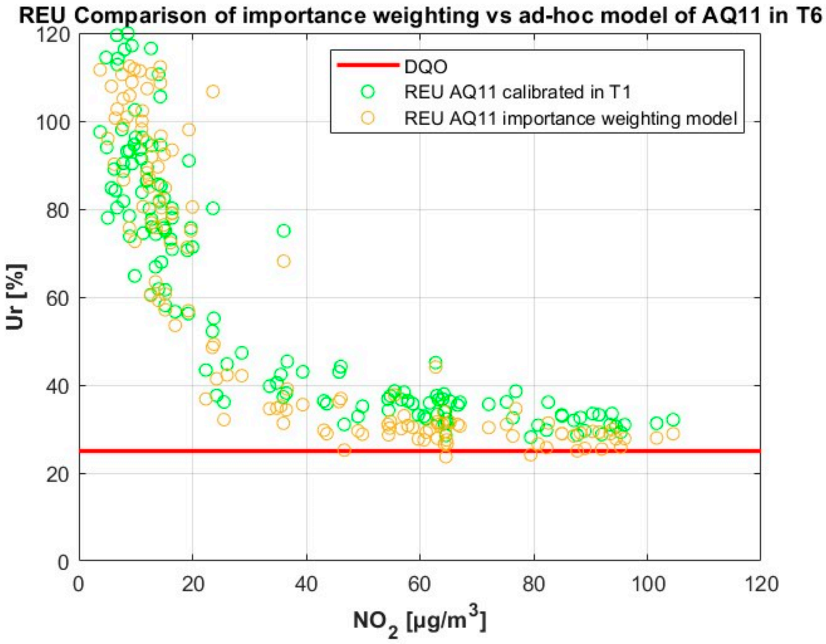

Figure S.3. 5 *Plot of Relative Expanded Uncertainties in T6 when AQ11 is re-calibrated with the importance weighted calibration model.*

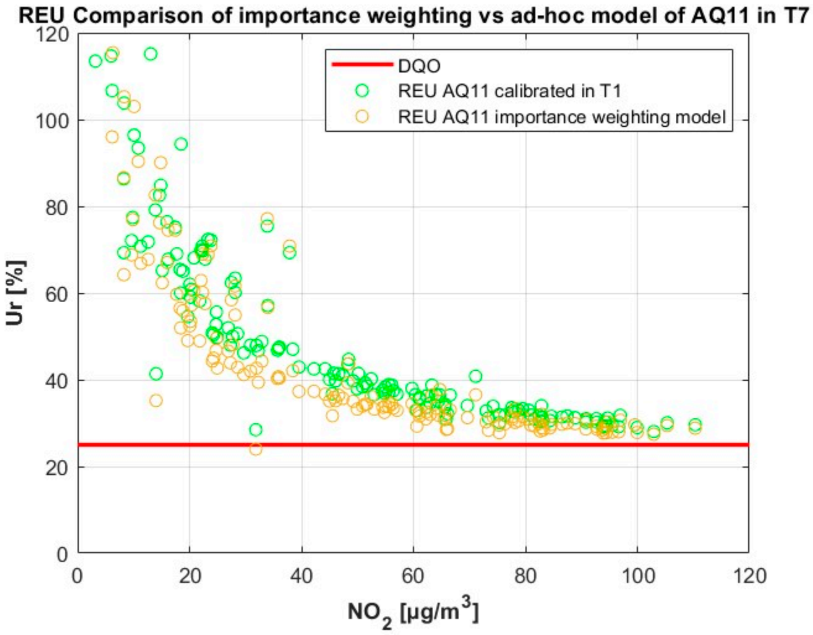

Figure S.3. 6 *Plot of Relative Expanded Uncertainties in T7 when AQ11 is re-calibrated with the importance weighted calibration model.*

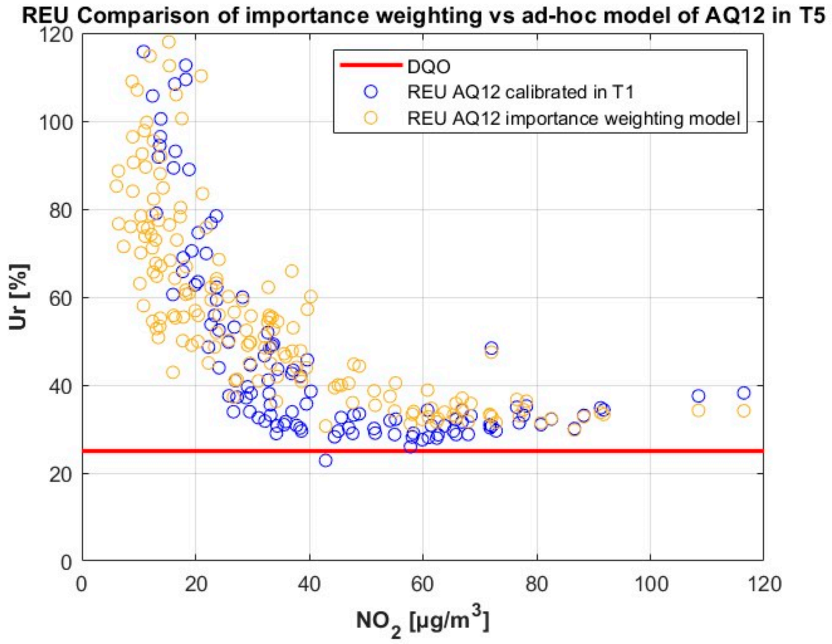

96

Figure S.3. 7 *Plot of Relative Expanded Uncertainties in T5 when AQ12 is re-calibrated with the importance weighted calibration model.* 97 98

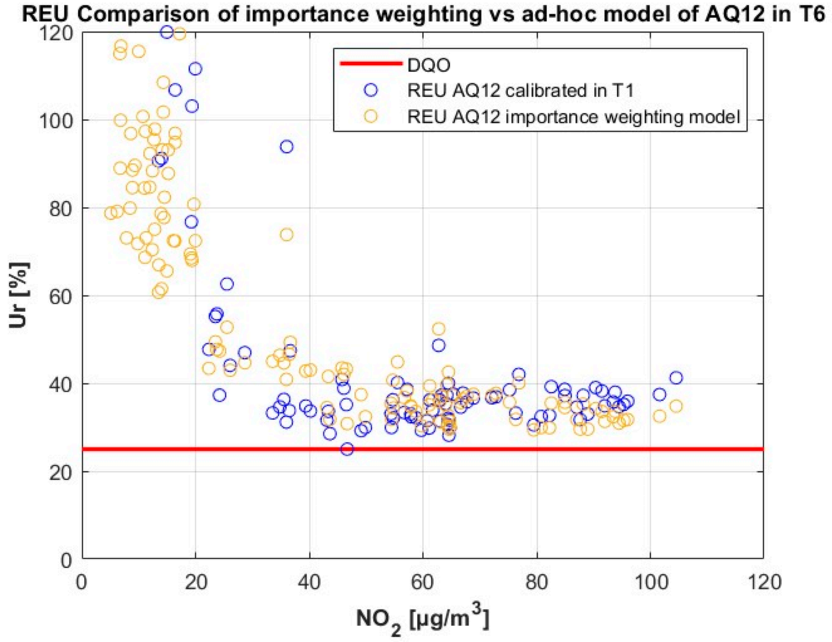

99

Figure S.3. 8 *Plot of Relative Expanded Uncertainties in T6 when AQ12 is re-calibrated with the importance weighted calibration model.* 100 101

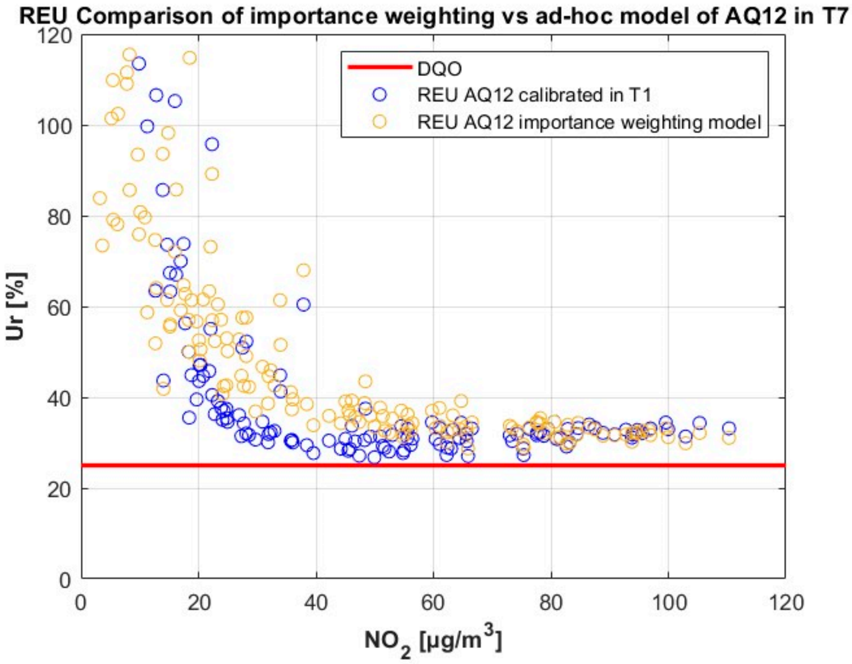

Figure S.3. 9 *Plot of Relative Expanded Uncertainties in T7 when AQ12 is re-calibrated with the importance weighted calibration model.*

S.4 REU plots of stacking ensemble calibration model

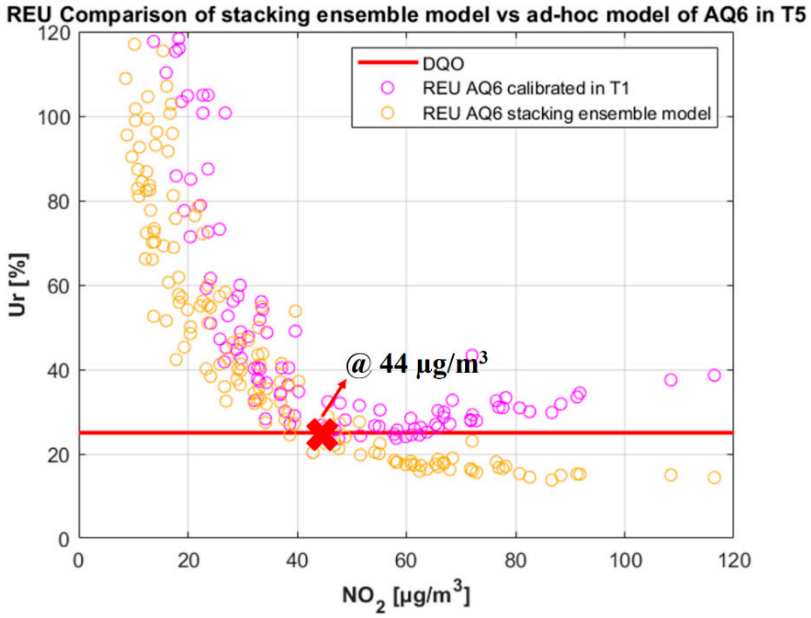

Figure S.4. 1 *Plot of Relative Expanded Uncertainties in T5 when AQ6 is re-calibrated with the stacking ensemble calibration model.*

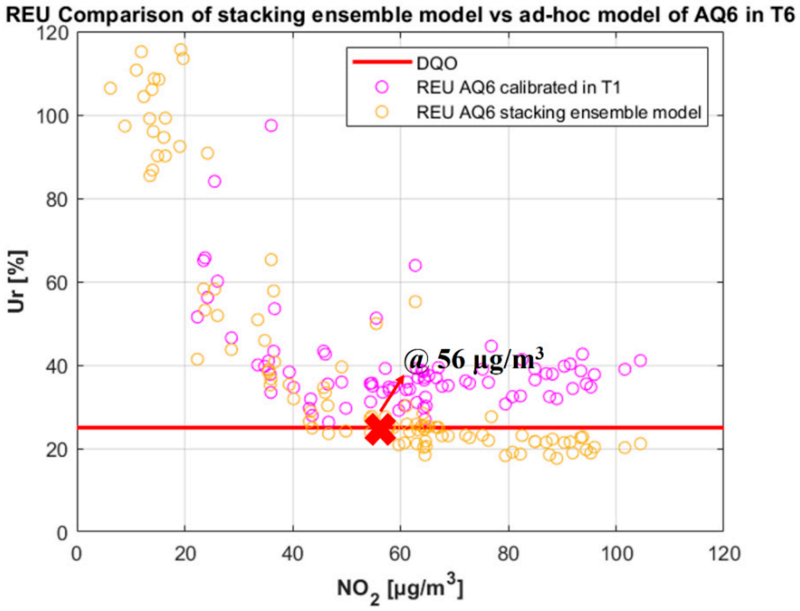

Figure S.4. 2 *Plot of Relative Expanded Uncertainties in T6 when AQ6 is re-calibrated with the stacking ensemble calibration model.*

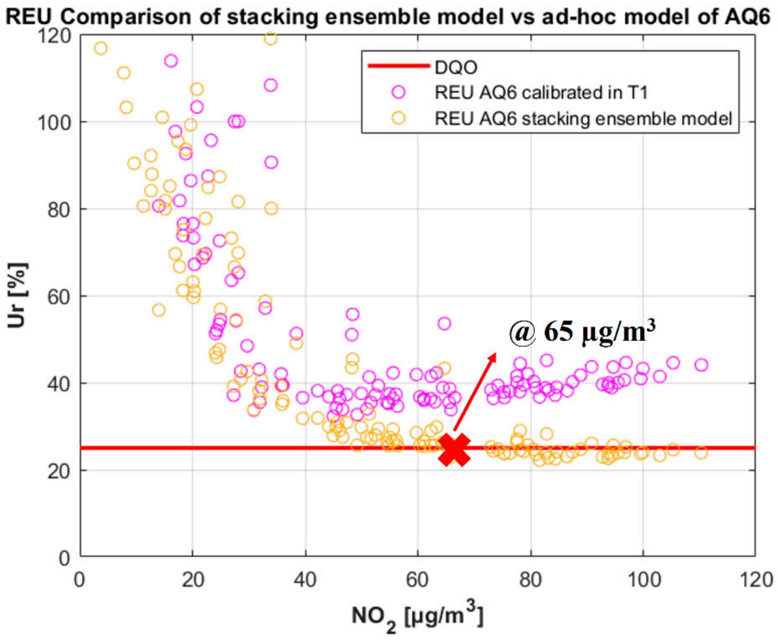

Figure S.4. 3 *Plot of Relative Expanded Uncertainties in T7 when AQ6 is re-calibrated with the stacking ensemble calibration model.*

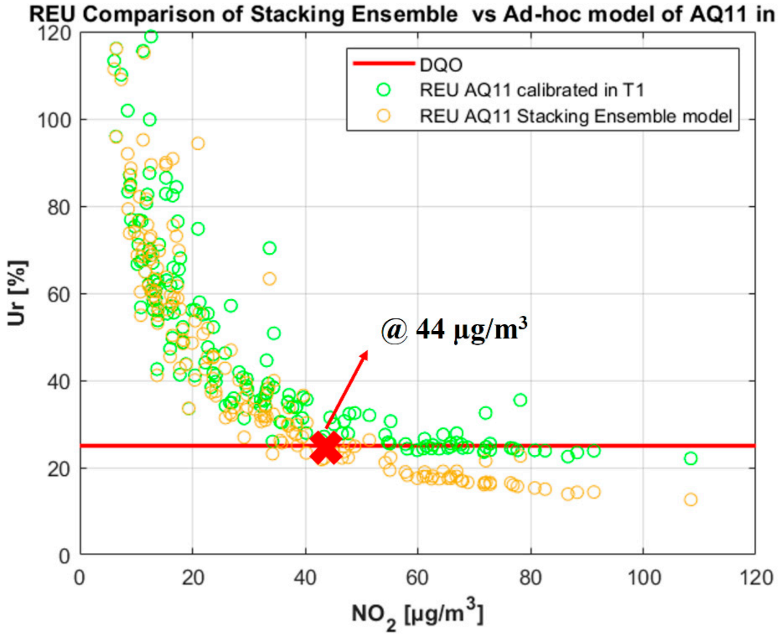

Figure S.4. 4 *Plot of Relative Expanded Uncertainties in T5 when AQ11 is re-calibrated with the stacking ensemble calibration model.*

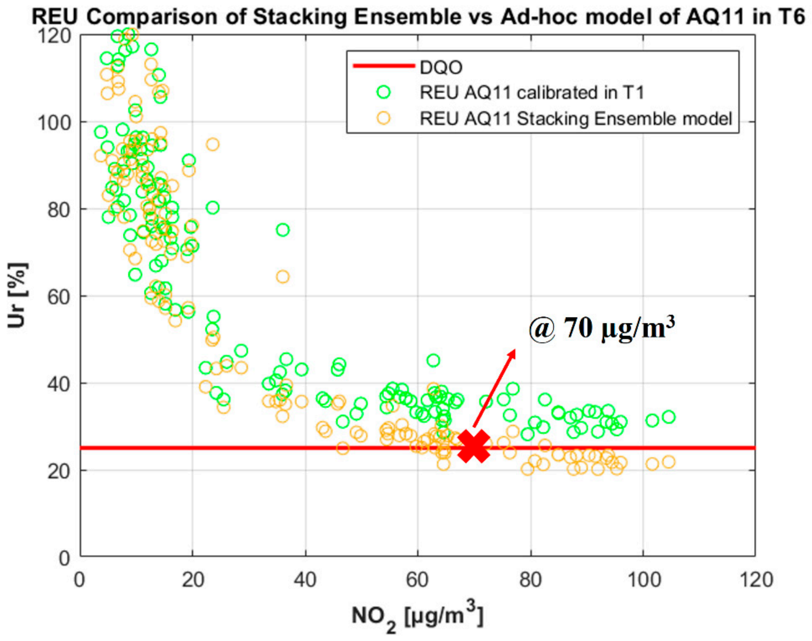

Figure S.4. 5 *Plot of Relative Expanded Uncertainties in T6 when AQ11 is re-calibrated with the stacking ensemble calibration model.*

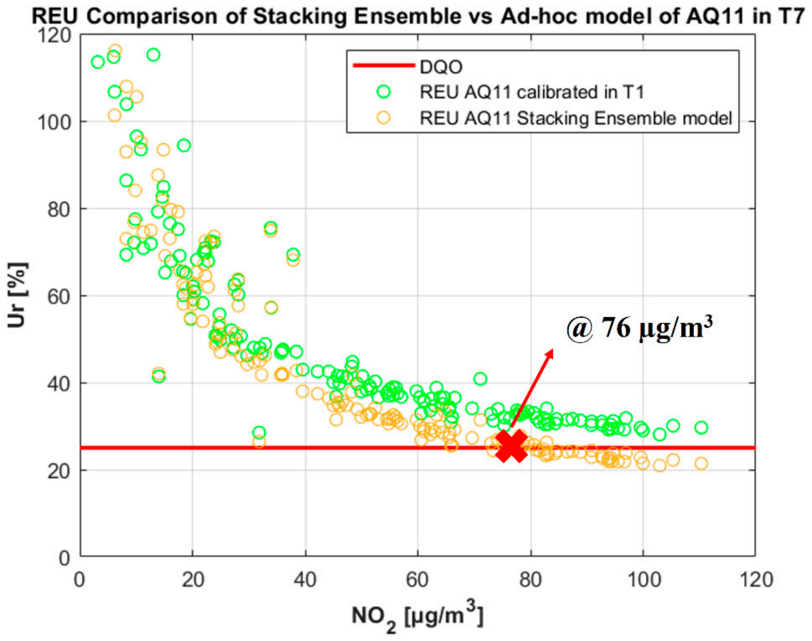

Figure S.4. 6 *Plot of Relative Expanded Uncertainties in T7 when AQ11 is re-calibrated with the stacking ensemble calibration model.*

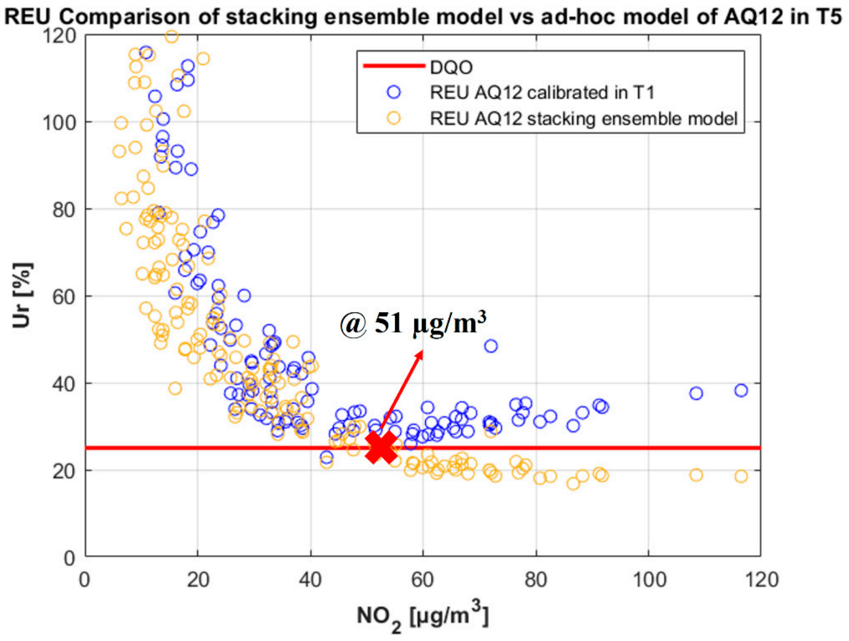

Figure S.4. 7 *Plot of Relative Expanded Uncertainties in T5 when AQ12 is re-calibrated with the stacking ensemble calibration model.*

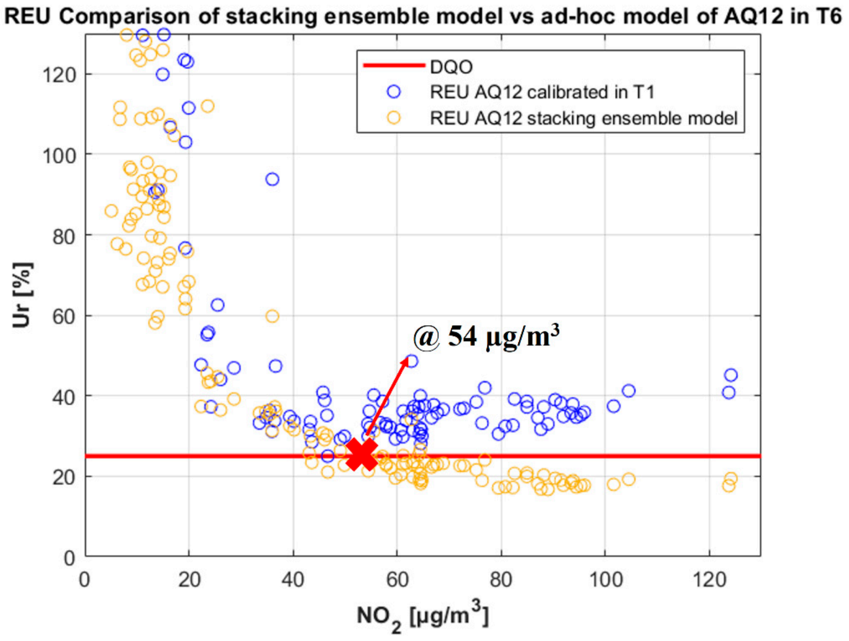

Figure S.4. 8 *Plot of Relative Expanded Uncertainties in T6 when AQ12 is re-calibrated with the stacking ensemble calibration model.*

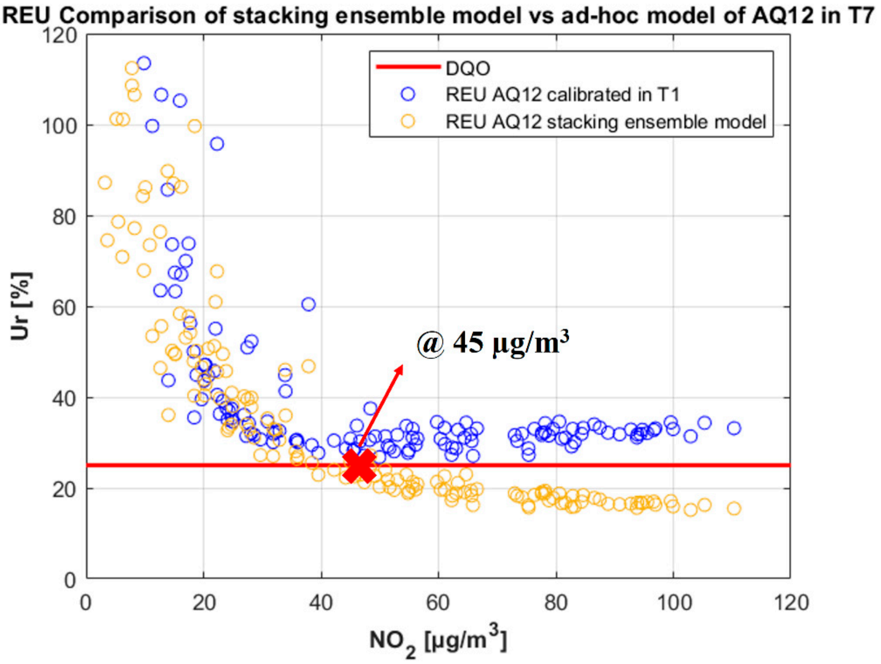

Figure S.4. 9 *Plot of Relative Expanded Uncertainties in T7 when AQ12 is re-calibrated with the stacking ensemble calibration model.*
